# Does bone debris in anterior cruciate ligament reconstruction really matter? A cohort study of a protocol for bone debris debridement

**DOI:** 10.1051/sicotj/2015014

**Published:** 2015-06-03

**Authors:** Mohamed A. Imam, Ashraf Abdelkafy, Feroz Dinah, Ajeya Adhikari

**Affiliations:** 1 South West London Elective Orthopaedic Centre Dorking Road Epsom KT18 7EG London, UK; 2 Orthopaedic Surgery and Traumatology Department, Faculty of Medicine, Suez Canal University Circular Road 41522 Ismailia Egypt

**Keywords:** Bone debris, Anterior cruciate ligament reconstruction, Debridement, Cyclops lesion, Protocol

## Abstract

*Background*: The purpose of the current study was to determine whether a systematic five-step protocol for debridement and evacuation of bone debris during anterior cruciate ligament reconstruction (ACLR) reduces the presence of such debris on post-operative radiographs.

*Methods*: A five-step protocol for removal of bone debris during arthroscopic assisted ACLR was designed. It was applied to 60 patients undergoing ACLR (Group 1), and high-quality digital radiographs were taken post-operatively in each case to assess for the presence of intra-articular bone debris. A control group of 60 consecutive patients in whom no specific bone debris protocol was applied (Group 2) and their post-operative radiographs were also checked for the presence of intra-articular bone debris.

*Results*: In Group 1, only 15% of post-operative radiographs showed residual bone debris, compared to 69% in Group 2 (*p* < 0.001).

*Conclusion*: A five-step systematic protocol for bone debris removal during arthroscopic assisted ACLR resulted in a significant decrease in residual bone debris seen on high-quality post-operative radiographs.

## Introduction

Anterior cruciate ligament reconstruction (ACLR) using either bone-patellar tendon-bone or hamstring grafts results in generation and accumulation of bone debris inside the knee joint, as well as the tibial and femoral tunnels. This occurs after drilling of the tibial and femoral tunnels and following notchplasty. In one study, bone debris were seen in post-operative radiographs in 63% of cases, and occasionally persisted for up to 6 months post-operatively [1]. Authors linked an increased prevalence and persistence of knee effusion in patients to the presence of visible debris on post-operative radiographs [1]. Other studies have suggested that early tunnel enlargement could result from bone necrosis and compacted bony debris created during and after drilling [2].

Furthermore, the bone dust and debris produced during ACLR may be responsible for generating osteophytes, which result in a false high diagnosis rate of osteoarthritis (OA) on subsequent follow-up radiographs [3]. OA was always believed to be an inevitable long-term consequence after ACLR, with prevalence ranging from 28% to 70% [4, 5]. Nevertheless, a recent meta-analysis showed that the prevalence of radiographic-proven knee OA after ACLR is less than what was commonly perceived [6].

Cyclops lesion is another complication that may be attributed to residual bone debris. Jackson and Schaefer first described it in 1990 in patients who had undergone ACLR with patellar tendon autograft [7]. Loss of motion, particularly knee extension, is a frequent cause of morbidity after ACLR. Cyclops lesions are the second most common cause of extension loss after ACLR, with a frequency of 1–9.8% [8].

The significance and fate of such bony debris is neither well documented nor understood. Designing a systematic protocol for the removal of bone debris in ACLR surgery has not been previously described.

The purpose of this study was to determine whether the use of a systematic protocol for the removal of bone debris during ACLR reduces the presence of bone debris seen on post-operative radiographs.

Hypothesis generation in the current study is that a systematic protocol would reduce the presence of bone debris after ACLR.

## Methods

This study was conducted as a prospective cohort study. A five-step protocol (see below) for removal of bone debris was designed and applied to 60 patients undergoing ACLR who were grouped as Group 1, while, a control group of another 60 patients in whom no specific bone debris removal protocol was applied were grouped as Group 2. High quality anteroposterior and lateral digital radiographs were performed pre- and post-operatively for all 120 patients included in the current study to assess the presence of intra-articular bone debris. Inclusion criteria were patients having no bony debris or loose bodies in their pre-operative radiographs. Exclusion criteria were patients having bony debris or loose bodies in their pre-operative radiographs.

**Figure 1.**
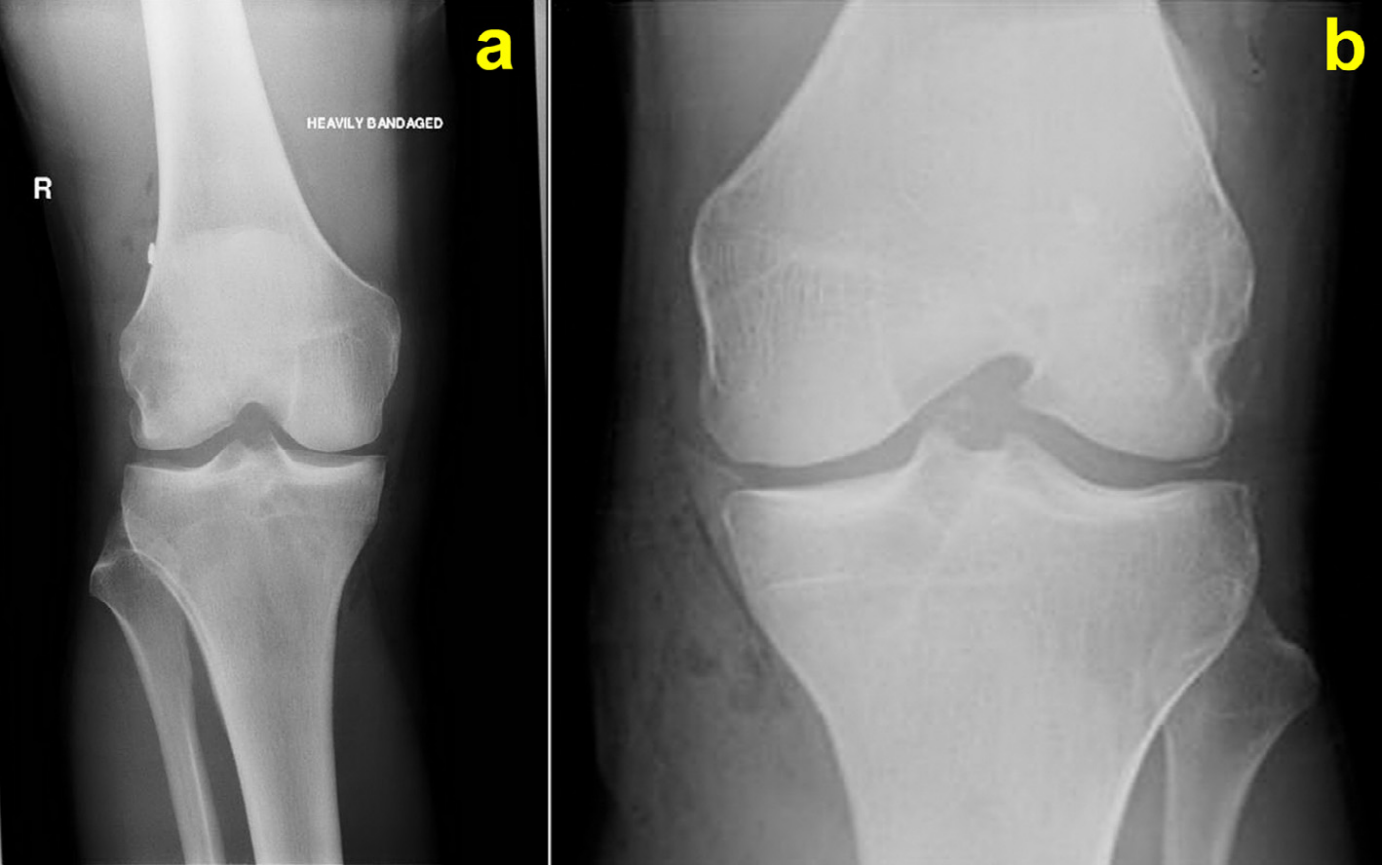
(a) Post-operative anteroposterior view of a right knee after ACLR showing no debris, (b) post-operative anteroposterior view of a left knee after ACLR showing debris.

The pre- and post-operative radiographs of all patients were reviewed and evaluated by one blinded expert in musculoskeletal radiology for the presence of debris to assess the effectiveness of the proposed protocol (Figures 1a and 1b).

### Surgical technique

#### Instruments

Standard knee arthroscopy set-up was used, including 4-mm 30° arthroscope, camera, tourniquet, recording system, shaver, pump, light source and monitor. No drainage cannula was used in any of the patients in either group.

#### Technique

Under general or spinal anaesthesia, the patient is placed in a supine position with the knee flexed at 90° at the side of the operating table or, alternatively, the thigh is placed in a leg holder and flexed freely at the removed foot of the table. Anterolateral, anteromedial, accessory medial are performed. The joint is inflated and maintained at pressure of 40 mm Hg.

### Protocol for debriding bone debris in five steps

#### 1. Debriding debris from posteromedial compartment

**Figure 2.**
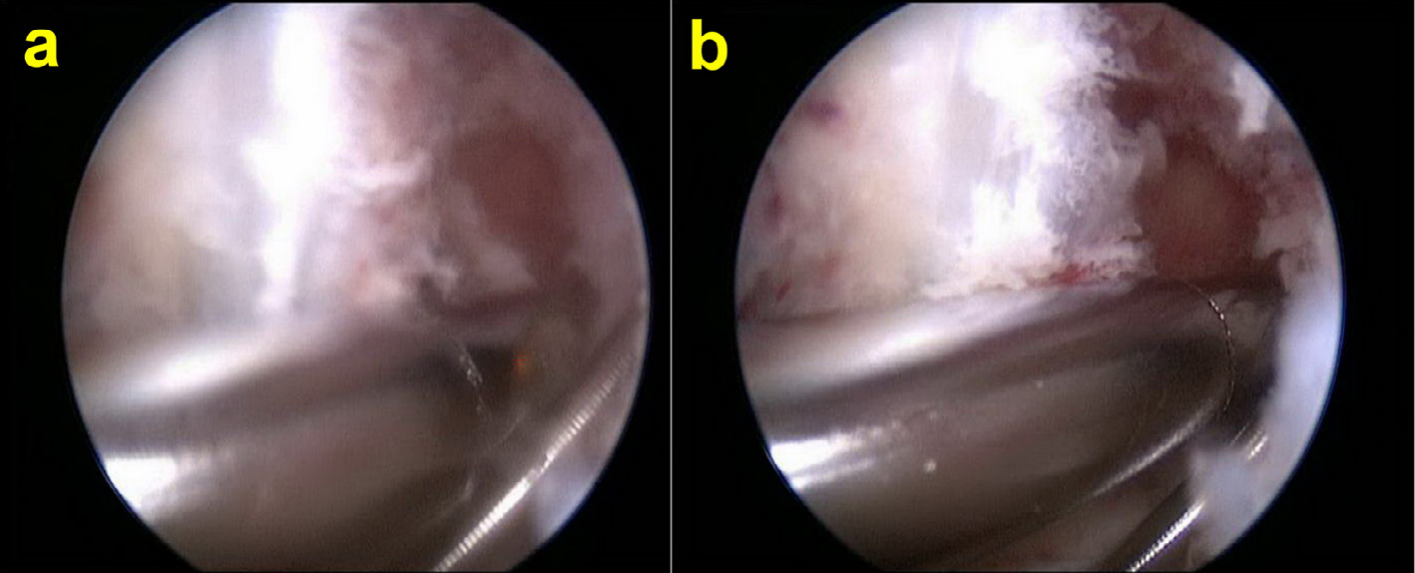
Debriding debris from posteromedial compartment. (a) Before debridement, (b) after debridement.

The shaver is introduced via the standard anteromedial portal and is used to debride the bone debris from the posteromedial joint space. This can be visualised by advancing the arthroscope further posteriorly into the notch using the Gilquist maneuver [9, 10] (introduction of the arthroscope through the intercondylar notch, under the posterior cruciate ligament and directly over the insertion of the posterior horn of the medial meniscus). This will allow a greater view of the posterior medial compartment. Debridement is continued around the notch (Figures 2a and 2b).

#### 2. Debridement of the tibial tunnel before graft advancement

**Figure 3.**
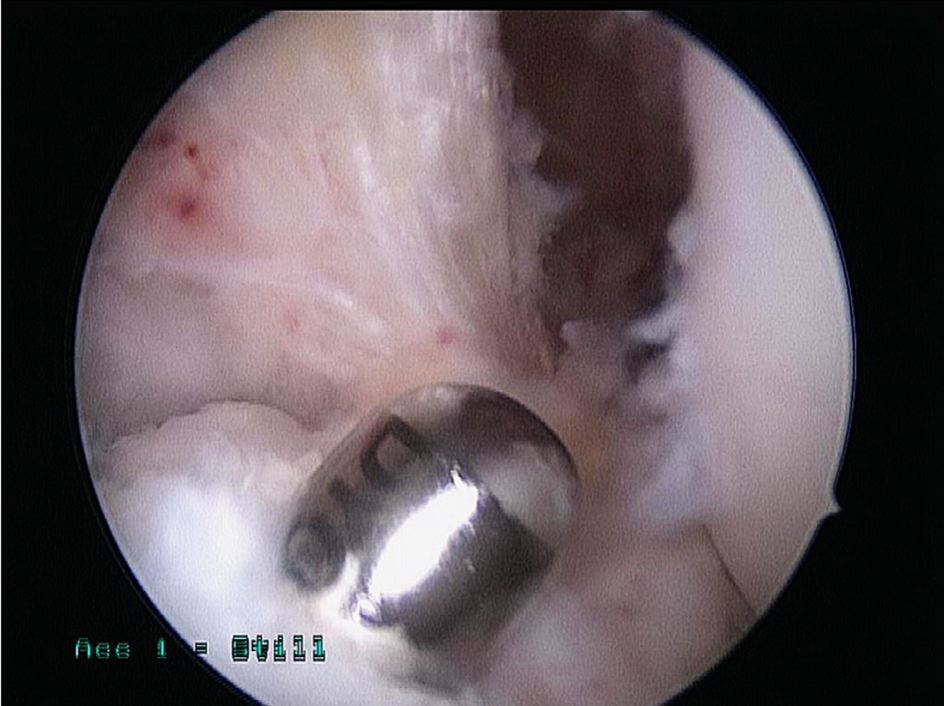
Debridement of debris from the tibial tunnel.

After drilling of the tibial tunnel, the shaver is inserted in the tibial tunnel from outside-to-in and used to debride all bone debris in the tibial tunnel (Figure 3). Then debridement of the posteromedial compartment is repeated again as described in the first step.

#### 3. Debridement of the femoral tunnel before graft advancement

**Figure 4.**
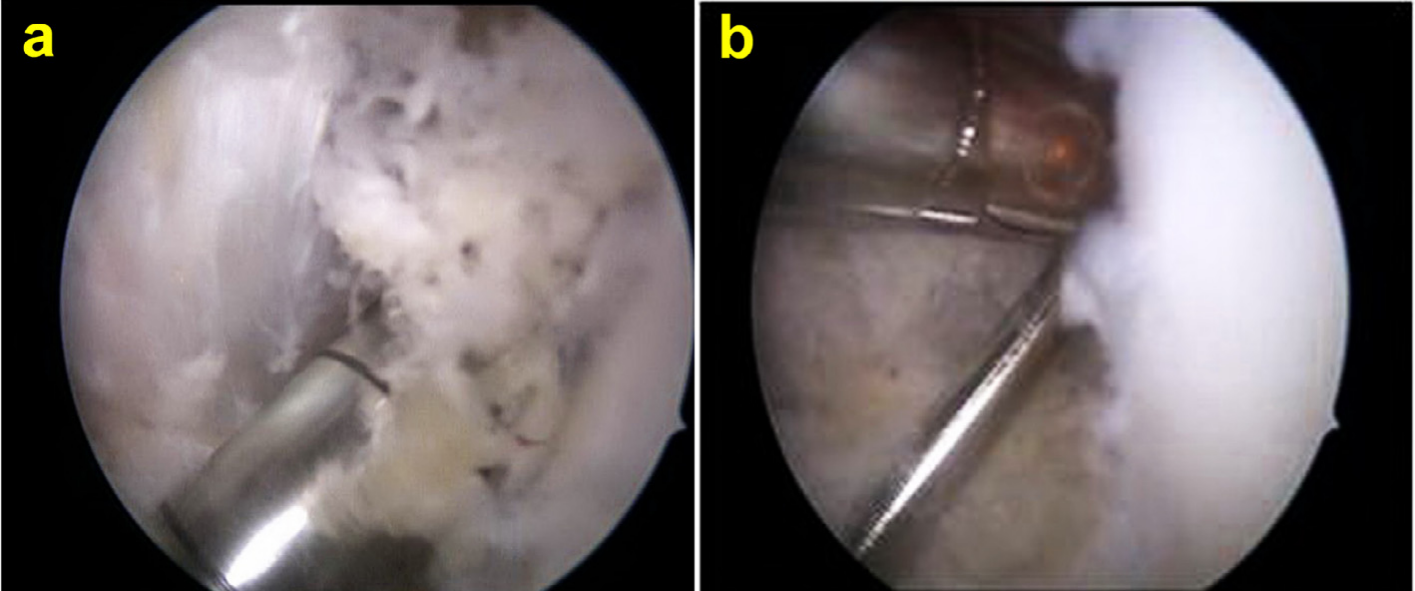
(a) Bone debris formed during femoral tunnel drilling, (b) debriding the femoral tunnel.

After drilling the femoral tunnel, the shaver is used to debride the bone debris at the femoral tunnel aperture, using the accessory medial or the standard anteromedial portals (Figures 4a and 4b). Then debridement of the posteromedial compartment is repeated again as described in the first step.

#### 4. Debridement of the suprapatellar pouch

**Figure 5.**
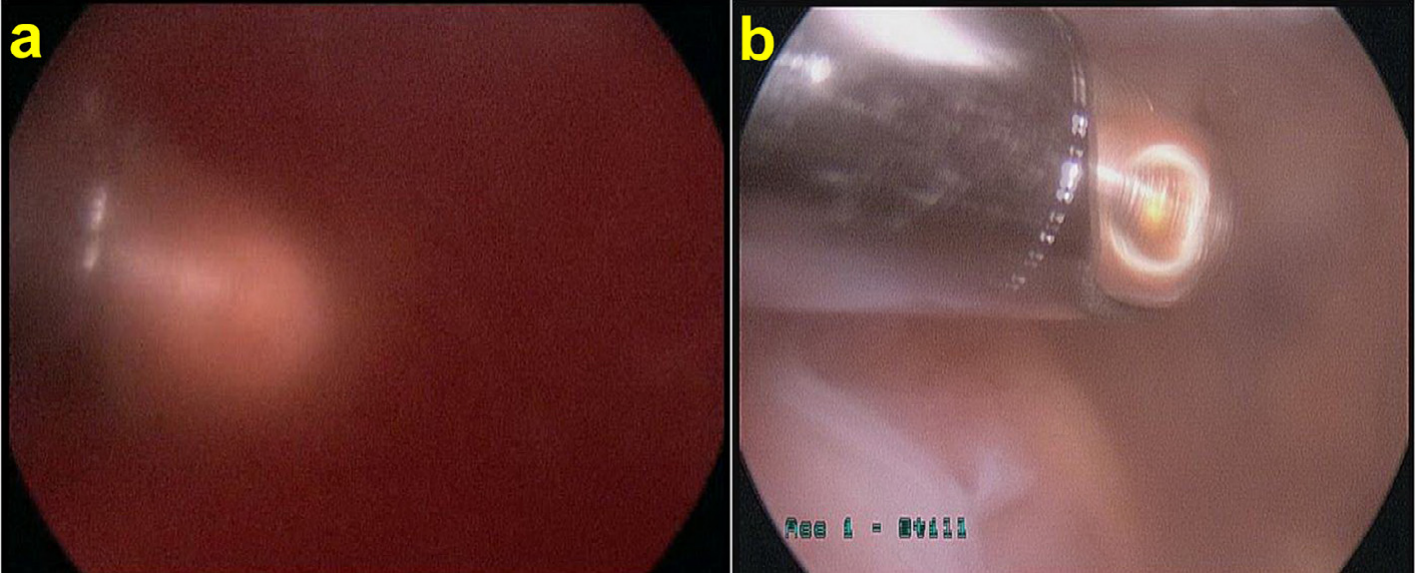
(a) Stormy appearance when the scope is inserted in the suprapatellar pouch, (b) debridement of the suprapatellar pouch using shaver with suction.

At the end of the procedure the scope is inserted in the suprapatellar pouch and the shaver is used to debride all the bone debris until a clear view is achieved (Figures 5a and 5b). Removal of bone debris from the suprapatellar pouch is an important step because by the end of the ACLR procedure, a lot of debris accumulates in it.

#### 5. Debriding the donor site

**Figure 6.**
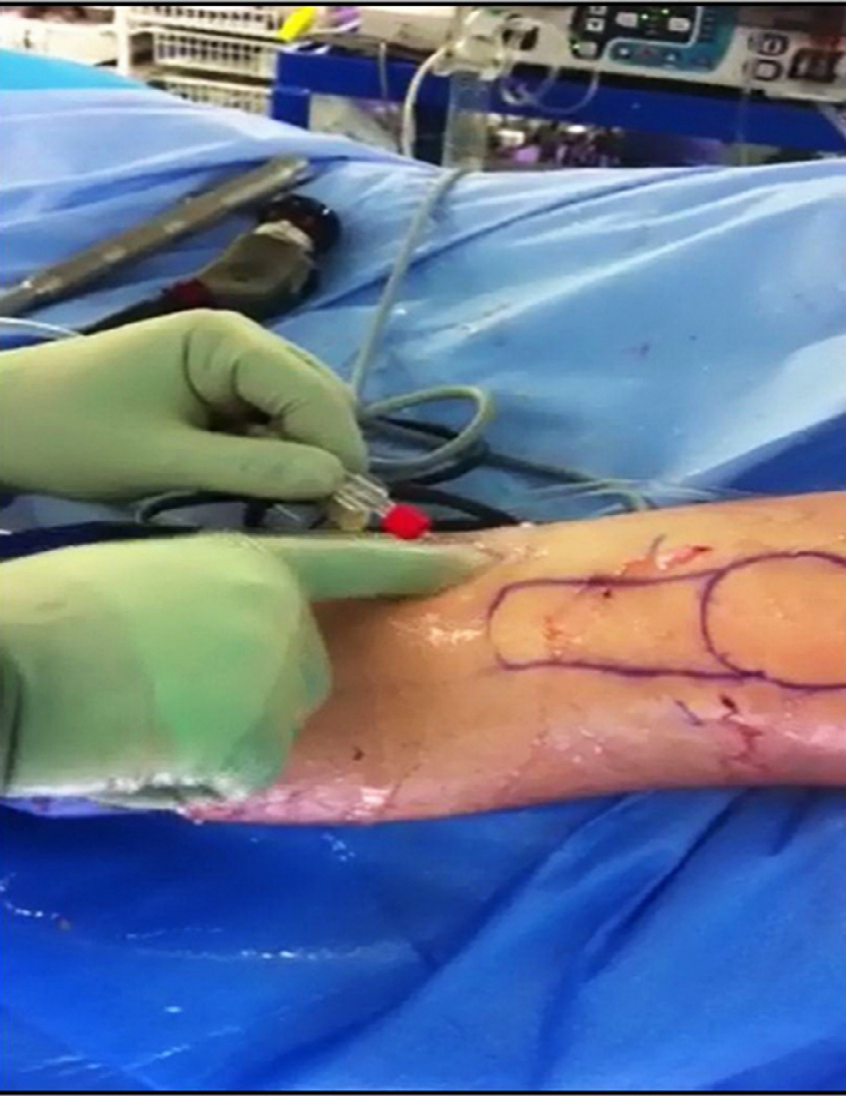
Debriding the tibial tunnel opening at the end of operation.

Prior to closure, the donor site is washed thoroughly to clean it from all debris (which might have accumulated after tibial drill bit retrieval) using the irrigation fluid (Figure 6).

### Statistical analysis

The initial set of analyses compared demographics, age and body mass index (BMI), of the two groups. These comparisons were performed using the unpaired *t*-test (SPSS 16.0 for Windows). The outcome of interest was the presence, or not, of bone debris on the post-operative radiographs. Due to the categorical nature of this outcome, the analysis was performed using the Chi-squared test (SPSS 16.0 for Windows).

## Results

There was no significant difference between the two groups as regarding age and BMI (*p* = 0.37, 0.94, respectively). In addition, no significant difference was found between the two groups in operative time (80.6 min for Group 1 vs. 78.4 min for Group 2, *p* = 0.32) (Table 1).

**Table 1.** Comparison between mean age, body mass index and operation time among both groups.

Variable	Protocol used [G1]	Protocol not used [G2]	*p*-value
	Mean (*SD*)	Mean (*SD*)	
Age	29.9 (8.6)	28.4 (8.7)	0.37
BMI	27.0 (5.0)	27.1 (4.6)	0.94
Operation time (minutes)	80.6 (13.2)	78.4 (11.3)	0.32

G1: Group 1, G2: Group 2, BMI: body mass index, *SD*: standard deviation.

A highly significant difference between the two groups (*p* < 0.001) was found regarding the presence of bone debris in the post-operative radiographs. Debris was far less common when the protocol was used, occurring in only 15% of patients, compared to 69% of patients when the protocol was not used. The relative risk of debris being present when the systematic protocol was not followed was calculated to be 4.5 (Table 2).

**Table 2.** Comparison between patients who showed debris and those who did not in post-operative radiographs among the two groups.

Use of protocol	No debris *N* (%)	Debris *N* (%)	Risk ratio (*) (95% CI)	*p*-value
Protocol used [G1]	51 (85%)	9 (15%)	0.22 (0.12-, 0.41)	<0.001
Protocol not used [G2]	19 (31%)	41 (69%)	1	

G1: Group 1, G2: Group 2, *N*: number, CI: confidence interval. (*) Expressed as risk of debris when protocol used compared to when not used.

## Discussion

The most noteworthy finding in the current study is that the protocol used for bone debris removal is effective in decreasing the amount of bone debris seen on post-operative radiographs; 15% in Group 1, compared to 69% in Group 2 (*p* < 0.001). Furthermore, comparing the two groups, the systematic protocol proposed did not significantly increase the operative time.

The bone debris seen on post-operative radiographs and generated during arthroscopic ACLR was studied by Wnorowski [1] who evaluated their incidence, effects and natural history. The author found no statistically significant difference in the presence of debris among different techniques. He also found that there is an increased prevalence and persistence of effusions in the debris group when compared to the non-debris group, even at 6 months post-operatively.

Jackson and Schaefer [7] reported on 13 cases out of 230 (5.7%) patients who underwent ACLR using patellar tendon autograft and had loss of full extension with an audible, palpable “clunk” with terminal extension. They proposed that the cyclops lesion was the result of a fibroproliferative process from accumulated bone debris. They also dramatically reduced the frequency of cyclops lesion formation by thoroughly debriding the tissue at the articular aspect of the tibial tunnel and by avoiding anterior positioning of the tibial tunnel.

Others also agreed that the development of these lesions is caused by bone debris and is stimulated by residual tissue at the rim of the tibial tunnel. They concluded that debris is pushed into the joint during placement of the graft and contribute to the cyclops lesion formation [11, 12]. Rubin et al. [13] documented the formation of inverted femoral cyclops after ACLR. They found that these patients presented with pain and stiffness 6 months after surgery. In another study, cyclops lesions were seen in the intercondylar notch anterior to and attached to the reconstructed ACL [14, 15].

Bone necrosis and compacted bony debris created during drilling were observed in the periphery of the tibial and femoral tunnels by a study conducted by Ugutmen et al. [2]. They emphasised that bone necrosis and compacted bony debris created during drilling would lead to early enlargement of both tunnels. The authors found that this sclerotic rim was observed in all patients, possibly due to necrotic tissue and compacted bony debris created during drilling. These rims were found to disappear after 3 weeks, leading to further tunnel enlargement.

Erdogan et al. [16] pointed out that extensive calcification of the patellar tendon after ACLR with central-third bone-patellar tendon-bone autograft could also be caused by bone debris.

In a study using single-photon emission computed tomography to evaluate OA after ACLR, the authors postulated that bone debris might be responsible for generating osteophytes and result in a falsely high diagnosis rate of early OA [3]. The incidence of OA after ACLR is still upsettingly high, with reports of nearly 50% of patients developing mild to moderate OA 6 years after surgery. Few studies have assessed the factors involved in the development of OA [3, 5, 17].

Advantages of the proposed protocol in the current study are: (1) Lowering the incidence of persistent effusion post-operatively, (2) decreasing the possibility of cyclops lesion formation, (3) decreasing tunnel enlargement, (4) only adds few minutes to the operation time.

Weak points of the current study are: (1) no controlled randomisation was applied for selection of patients, (2) short-term follow-up.

Strong points of the current study: (1) a large number of patients included in the current study (120 patients), (2) the presence of a control group for comparison.

## Conclusion

A systematic five-steps protocol for bone debris removal during arthroscopic assisted ACLR results in a significant decrease in residual bone debris seen on post-operative radiographs.

## Conflict of interest

The authors report that they have no conflicts of interest in the authorship and publication of this article.
